# AGING BODY AS CONTEXT: THE ROLE OF INTEROCEPTIVE AGING IN MID- AND LATE-LIFE EMOTION

**DOI:** 10.1093/geroni/igac059.1443

**Published:** 2022-12-20

**Authors:** Jennifer MacCormack

**Affiliations:** University of Virginia, Charlottesville, Virginia, United States

## Abstract

Interoceptive sensations (e.g., racing heart, clenched gut) are often closely tied to emotion. Older adults tend to exhibit reduced sensitivity and awareness of their interoceptive sensations relative to younger adults (e.g., Khalsa et al., 2009; Mikkelsen et al., 2019; Murphy et al., 2019), yet the emotional implications of such interoceptive differences remain unclear. Herein, I present two behavioral cross-sectional studies (N=350) documenting age differences in how adults (18-75 yrs) link their interoceptive sensations to emotions (Study 1: cognitive behavioral task; Study 2: experience sampling). Results reveal that from midlife into late life, coherence between interoceptive sensations and emotions increasingly weakens, both behaviorally and in self-reports. Age effects are most prominent for high arousal sensations and states. These findings provide converging evidence with recent neuroimaging results (MacCormack et al., 2020) showing the importance of interoceptive aging and related shifts in physiological arousal as potential pathways by which emotions transform across adulthood.

